# 1327. Nontyphoidal *salmonella* infection in children and adolescent; A retrospective, multicenter study in Korea

**DOI:** 10.1093/ofid/ofac492.1157

**Published:** 2022-12-15

**Authors:** Je Hee Shin, Sung Min Lim, Joon-sik Choi, Ji Hong Kim, Jong Gyun Ahn, Ji-Man Kang

**Affiliations:** Severance Children’s Hospital, Yonsei University College of Medicine, Seoul, Seoul-t'ukpyolsi, Republic of Korea; Severance Children’s Hospital, Yonsei University College of Medicine, Seoul, Seoul-t'ukpyolsi, Republic of Korea; Yongin Severance Hospital, Yonsei University College of Medicine, Yongin, Korea, Yongin, Kyonggi-do, Republic of Korea; Severance Children’s Hospital, Yonsei University College of Medicine, Seoul, Seoul-t'ukpyolsi, Republic of Korea; Severance Children’s Hospital, Yonsei University College of Medicine, Seoul, Seoul-t'ukpyolsi, Republic of Korea; Severance Children’s Hospital, Yonsei University College of Medicine, Seoul, Seoul-t'ukpyolsi, Republic of Korea

## Abstract

**Background:**

Non-typhoidal *salmonella* (NTS) infections usually have a self-limiting course, but can cause invasive NTS (iNTS) diseases, including bacteremia, meningitis, osteomyelitis or other focal infection. However, information on NTS infections in children is scarce in Asian countries. We investigated the differences in clinical features and antimicrobial susceptibility patterns of NTS infections in Korean children.

**Methods:**

From November 2006 to May 2021, we collected NTS cases isolated from patients under the age of 20 at three Severance Hospitals (Sinchon, Gangnam, and Yongin) in Korea. All NTS cases were extracted through the Severance Clinical Research Analysis Portal. NTS cases isolated from the genitourinary tract were excluded. Clinical data were collected through chart review.

**Results:**

A total of 837 isolates were identified from 637 patients. 22 patients were excluded, and a 615 patients were included in the study. The median age at NTS infection was 4.6 years (IQR, 2.4-7.9 years), and those under 5 years of age accounted for 54.1% of all cases. The male to female ratio was 1.6:1 and approximately 15.8% (n=97) had at least one comorbidity. By clinical diagnosis, enterocolitis was the most common with 543 cases (88.3%), followed by bacteremia without local sign in 11 cases (1.8%), osteomyelitis in 7 cases (1.1%). The iNTS group (n=68) did not have any significant differences in age of onset, presence of fever, white blood cells, absolute neutrophils, and C-reactive protein levels, but the frequency of diarrhea (67.6% vs. 89.9%, *p*<*0.001*) was less than the non-invasive NTS group (n=547). Extra-intestinal symptoms were more common in the iNTS group than in the non-invasive group (30.9% vs. 14.3%, *p=0.003*), and the length of hospital stay (8.65±6.4 days vs. 5.13±3.3 days, *p*=*0.001*) were significantly longer in the iNTS group. Notably, 2 out of 7 patients with NTS osteomyelitis showed a poor clinical response even with cefotaxime-sensitive antimicrobial susceptibility results. Antimicrobial susceptibility pattern between the group was shown in **Figure 1**.

Antimicrobial susceptibility pattern between the group

**Figure:**
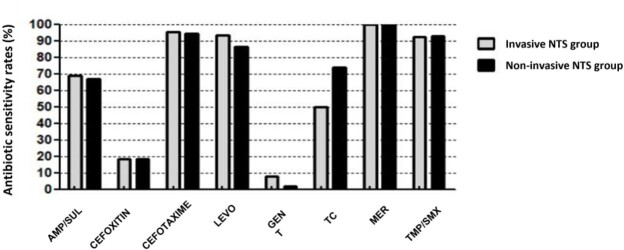

**Conclusion:**

Children with iNTS infection showed a relatively severe clinical course compared to children with non-iNTS infection. Further studies on the epidemiology and characteristics of invasive NTS infection are needed.

**Disclosures:**

**Joon-sik Choi, MD, MS**, Ministry of Trade, industry and Energy, Republic of Korea: Grant/Research Support.

